# Isolated Splenic Metastases from Renal Cell Carcinoma 11 Years after Surgery

**DOI:** 10.1155/2019/7480479

**Published:** 2019-09-24

**Authors:** Davi dos Santos Romao, Natally Horvat, Marianne Castro Gonçalves, Emerson Shigueaki Abe, Rodrigo Blanco Dumarco, Publio Cesar Cavalcante Viana, Marcel Cerqueira Cesar Machado

**Affiliations:** ^1^Department of Radiology, Hospital Sírio-Libanês, São Paulo, Brazil; ^2^Department of Pathology, Hospital Sírio-Libanês, São Paulo, Brazil; ^3^Department of Surgery, Hospital Sírio-Libanês, São Paulo, Brazil

## Abstract

Splenic metastases are rare and usually occur in cases of disseminated disease. We report a case of a patient who had isolated splenic metastases with a previous history of left nephrectomy due to a renal cell carcinoma 11 years before. The aim of this report is to describe the case and review the literature of isolated splenic metastases due to renal carcinoma. This case emphasizes the importance of considering splenic metastatic disease even after many years of diagnosis of renal cell carcinoma.

## 1. Introduction

Splenic tumors, primary or metastatic, are rare and most commonly caused by haematological malignancies. The most frequent primary tumors that metastasize to the spleen are malignant melanoma, breast cancer, and lung and ovarian carcinoma [1], and it is usually a part of a disseminated metastatic disease. Metastatic disease is common in renal- cell carcinoma (RCC), and it is frequent to the lungs, bone, liver, adrenal glands, stomach, pancreas, brain, and contralateral kidney [2]. However, isolated splenic metastases from RCC are extremely rare. To the best of our knowledge, there are less than 20 cases of isolated splenic metastases from RCC reported in the literature [1, 3–16].

The aim of this report is to describe a case of an adult patient with a previous history of left nephrectomy due to an RCC who had isolated splenic metastases 11 years after the primary renal tumor.

## 2. Case Report

A 48-year-old man complained of intermittent pain in the left flank that started 4 months ago, and it was associated with nausea. Physical examination was normal, except for mild discomfort in the upper abdomen. Laboratory studies were also within normal limits.

**Figure 1:**
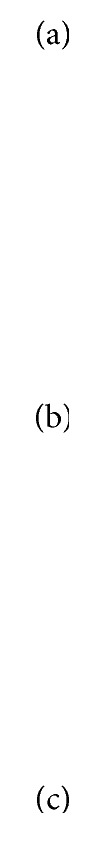
Contrast-enhanced MRI of the abdomen on initial staging of the renal tumor. (a) Coronal T1-weighted image and (b) axial T2-weighted image demonstrated a left renal mass in the upper pole of the left kidney with cystic areas (arrows).

He had a previous history of RCC (Figure 1) when he underwent left nephrectomy 11 years before (pT2, pV0, and pN0; Fuhrman grade 1). The RCC infiltrated beyond the renal capsule, but it was contained within the Gerota's fascia. Pathology showed a subtype of clear cell RCC. He also had a history of B-cell non-Hodgkin lymphoma on the scalp 8 years earlier, which was treated with surgery and radiotherapy.

**Figure 2:**
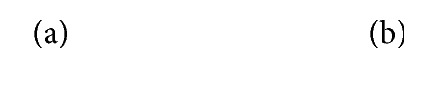
Contrast-enhanced CT of the abdomen 11 years after the left nephrectomy. (a) Coronal and (b) axial images showed two solid-cystic splenic masses (arrows) with heterogeneous enhancement, infiltrating the diaphragm (arrowhead).

**Figure 3:**
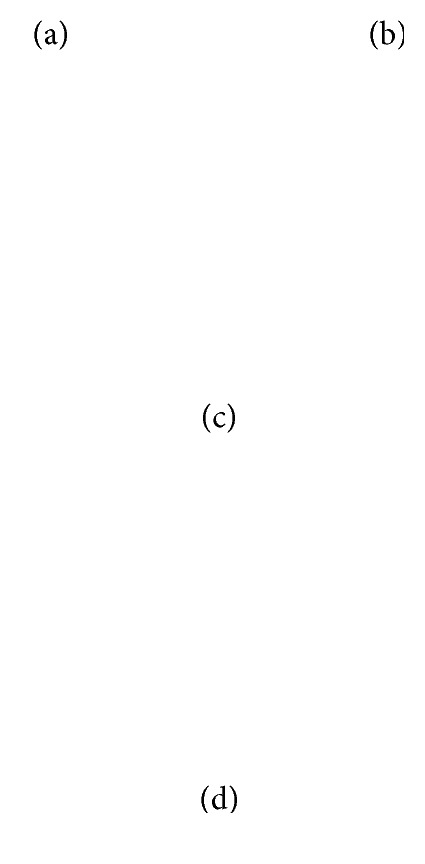
Contrast-enhanced MRI of the abdomen 11 years after the left nephrectomy. (a) Axial T2-weighted image and (b) axial postcontrast T1-weighted image depicted two solid-cystic splenic masses (arrows), infiltrating the diaphragm (arrowhead).

**Figure 4:**
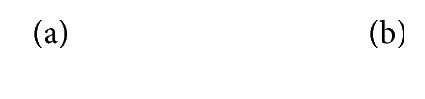
Positron emission computed tomography (PET/CT) scan of the whole body showed two splenic masses (arrows) indistinct on CT without intravenous contrast (a), with increased FDG uptake (SUV 9,0) (b), and without other suspicious lesions (c).

Abdominal computed tomography (CT) with intravenous contrast was requested to evaluate his symptoms. CT scan demonstrated two heterogeneous hypovascular splenic masses with necrotic areas, situated in the posteroinferior portion. The lesions extended through the splenic capsule and abutted the left diaphragm (Figure 2). Magnetic resonance imaging (MRI) was requested for further evaluation and demonstrated the splenic masses, with lobulated contours, heterogeneous signal intensity, and central necrotic areas (Figure 3). The splenic masses measured 4.5 cm and 5.8 cm. Additionally, a whole-body positron emission tomography scan (PET/CT) was performed, and it revealed an increase in FDG uptake within the splenic mass (SUV: 9.0), without other suspicious lesions (Figure 4).

**Figure 5:**
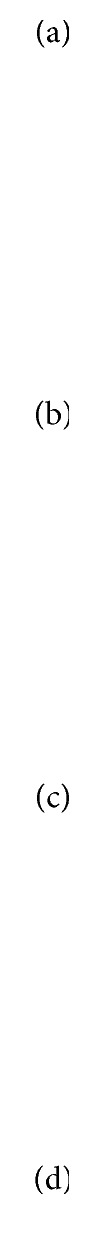
Surgical specimen of splenectomy demonstrated two lobulated yellowish lesions within the spleen (a, b). A portion of the left diaphragm was also resected due to the adhesion process (c, arrowhead).

**Figure 6:**
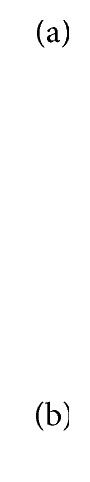
Detail of the neoplasm, composed of nests and solid sheets of cells with abundant and clear cytoplasm. The nuclei are enlarged with some conspicuous nucleoli (H&E 200x).

**Figure 7:**
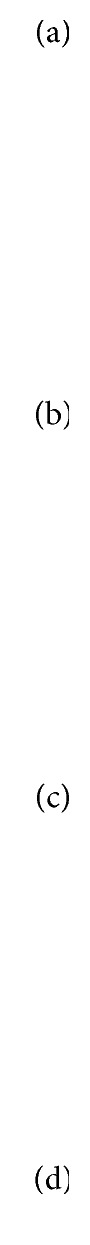
The neoplastic cells expressed CK8/18 (a), CD10 (b), PAX8 (c), and vimentin (d), being consistent with metastasis of clear cell renal carcinoma.

The patient underwent splenectomy. On pathological examination, the spleen had two yellowish lesions (Figure 5), measuring up to 6.0 cm. On histopathological analysis, areas of tumoral necrosis, angiolymphatic invasion, and focal involvement of the diaphragm were observed. Immunohistochemistry assay was positive for CK8/18, CD10, PAX8, and vimentin, being consistent with metastases of clear cell RCC (Figures 6 and 7). It is important to note that PAX8 fixation was positive for nuclear staining although weak likely due to poor splenic parenchyma fixation (Figure 7(c)). The patient is in his 3rd month after surgery with complete resolution of his symptoms and in a good general health condition.

## 3. Discussion

Splenic metastases are rare and usually occur in cases of disseminated disease. Isolated splenic metastases from RCC are extremely rare. This rarity might be explained by several theories related to the anatomical, histological, and immunological characteristics of the spleen. The theories include the following: (a) sharp angle of the splenic artery, which may impair the migration of the tumor emboli to the spleen; (b) physiological rhythmic contractile activity of the spleen, squeezing out the tumor emboli; (c) absence of afferent lymphatics, which could bring tumor cells; and (d) high concentration of lymphoid tissue in the spleen, resulting in antitumor activity [3].

There is an estimation that 18% of patients with RCC have metastasis at diagnosis (synchronous metastasis), and after surgical excision, 20%–30% of patients with localized tumors experience relapse [17]. The majority of recurrences (85%) occur within the first 3 years of follow-up. The most common sites of metastatic disease from RCC are the lungs, bone, lymph node, and liver [18].

Isolated spleen metastases may be underestimated, considering that the patients are often asymptomatic or oligosymptomatic. However, recently, more cases of isolated splenic metastases have been diagnosed due to the widespread use of diagnostic imaging modalities. Splenic metastases originating from RCC may be detected synchronously with the renal tumor in 30% of the cases [9] or soon after its diagnosis. However, they can also arise several years after the treatment of the primary tumor as presented in our case.

Isolated splenic metastases from RCC are extremely rare, and to the best of our knowledge, up to now, there are less than 20 cases reported in the literature [1, 3–16].  Table 1 summarizes the main studies that reported the cases of splenic metastases from RCC [1, 3–16].

The average age of patients with splenic metastases from RCC was 62 years, ranging from 29 to 75 years, and the majority were men (11/14, 78%). The majority of the renal primary tumors was in the left kidney (10/14, 71%), which led to a hypothesis of direct spread of the tumor cells rather than haematogenous metastasis; however, the Gerota's fascia was intact in the majority of the tumors [10] similar to our case which suggests that it is an isolated metastasis rather than a case of local recurrence. Patients with splenic metastases from RCC were asymptomatic (6/14, 43%) or had abdominal pain or constitutional symptoms, such as fever, fatigue, weight loss, and anemia. The splenic renal metastases were diagnosed at the same time of the primary tumor in 5/14 (36%) cases (synchronous metastases). Among the metachronous cases, the metastasizing interval between the primary renal tumor and the splenic metastasis varied from 2 months to 22 years. Among the cases that specified the subtype of RCC, the following subtypes were demonstrated: clear cell and papillary. With regards to imaging features, the vast majority of the splenic lesion was solitary and demonstrated a heterogeneous pattern of enhancement on CT and MRI. Three patients underwent PET/CT; the splenic lesion of one patient had no metabolic activity, and in the other 2 patients, the splenic lesions were hypermetabolic.

As we can observe, imaging modalities demonstrated a pivotal role in the detection of the splenic lesions. Although not all lesions were PET/CT avid, it is a useful tool to investigate other lesions in other organs, which may change the management in these cases.

According to the literature, there is a survival benefit to an aggressive approach and a complete surgical resection of the primary tumor and metastatic sites, when technically feasible, may offer the best opportunity for cure [19]. The vast majority of the patients described in the literature underwent surgical resection. Among the reports that described the patient outcome, 2 patients died 5 and 12 months after the detection of splenic metastases and 9 patients were alive up to 2 years of follow-up.

This case report emphasizes the importance of considering splenic metastases from RCC even in patients without disseminated disease and many years after the primary tumor diagnosis. This case also stresses the need of extended follow-up since early diagnosis and treatment may improve patients' outcome and reduce morbidity, especially considering that complete surgical resection is the main curative treatment.

## Figures and Tables

**Table 1:** Summary of cases of isolated splenic metastasis found in the literature.

Author	Age/sex	Metastasizing time	Primary (kidney)	Symptoms	Outcome	Histology type	Imaging findings	Met size
Strum [4]	59 y/M	22 y	Right	CS	Dead (5 mo)	NS-RCC	ND	ND
Ishida et al. [5]	50 y/M	7 y	Left	—	Alive (6 y)	NS-RCC	US: one solid-cystic mass with echogenic margin	4.0 cm
Nabi et al. [14]	50 y/F	(synchronous)	Left	P	Alive (6 mo)	NS-RCC	CT: one solid-cystic hypoattenuating splenic mass	ND
Kugel et al. [6]	72 y/M	2 y	Left	CS	Dead (1 y)	CC-RC	CT: one solid-cystic hypoattenuating splenic mass	8.0 cm
McGregor et al. [7]	65 y/M	(synchronous)	Left	CS, P	ND	P-RCC	CT: one hypoattenuating, heterogeneously enhancing, splenic mass	8.0 cm
Shuck-Bello et al. [15]	74 y/M	15 y	Right	—	ND	CC-RC	CT and MRI: one hypoattenuating (CT), hypervascular splenic mass with calcifications	ND
Ielpo et al. [9]	82 y/M	14 y	Left	—	Alive (1 y 3 mo)	NS-RCC	(i) CT: one solid-cystic hypovascular mass (ii) PET/CT: no metabolic activity	6.0 cm
Moir et al. [10]	70 y/F	11 mo	Left	CS	Alive (2 y)	CC-RC	CT: one solid-cystic hypervascular splenic mass	7.0 cm
Nunes et al. [11]	60 y/F	5 y	Left	—	Alive (6 mo)	CC-RC	(i) CT and MRI: one small hypervascular splenic nodule (ii) MRI (6 mo later): heterogeneous hypervascular mass with central necrosis	1.0 cm–4.0 cm
Hardikar [16]	29 y/M	(synchronous)	Left	—	Alive (2 y)	CC-RC	CT: one hypoattenuating splenic mass	ND
Zhang et al. [12]	67 y/M	2 y	Left	CS	Alive (5 mo)	NS-RCC	(i) CT w/o contrast: one isoattenuating splenic mass (ii) PET/CT: one hypermetabolic mass	11.4 cm
Grewal et al. [1]	53 y/M	2 mo	Left	—	ND	CC-RC	PET/CT: multiple splenic lesions, one hypermetabolic	ND
Liu et al. [13]	75 y/M	(synchronous)	Right	CS	Alive (1 y 4 mo)	P-RCC	(i) CT: one indeterminate hypodensity (ii) CT (9 mo later): heterogeneous splenic mass	0.6–9.9 cm
Romão, 2019	48 y/M	11 y	Left	P	Alive (2 mo)	CC-RCC	(i) CT and MRI: two solid-cystic hypervascular masses (ii) PET/CT: two hypermetabolic masses	5.8 cm

CS: constitutional symptoms, such as weight loss and fatigue; P: pain; ND: not described; NS-RCC: nonspecified renal cell carcinoma; CC-RC: clear cell renal carcinoma; P-RCC: papillary renal cell carcinoma.
